# Cutaneous Chronic Graft-Versus-Host Disease Does Not Have the Abnormal Endothelial Phenotype or Vascular Rarefaction Characteristic of Systemic Sclerosis

**DOI:** 10.1371/journal.pone.0006203

**Published:** 2009-07-09

**Authors:** Jo Nadine Fleming, Howard M. Shulman, Richard A. Nash, Pamela Y. Johnson, Thomas N. Wight, Allen Gown, Stephen M. Schwartz

**Affiliations:** 1 Department of Pathology, University of Washington, Seattle, Washington, United States of America; 2 Clinical Research Division, Pathology Section, Fred Hutchinson Cancer Research Center, Seattle, Washington, United States of America; 3 Clinical Research Division, Fred Hutchinson Cancer Research Center, Seattle, Washington, United States of America; 4 Benaroya Research, Seattle, Washington, United States of America; 5 Phenopath Laboratories, Seattle, Washington, United States of America; Health Canada, Canada

## Abstract

**Background:**

The clinical and histologic appearance of fibrosis in cutaneous lesions in chronic graft-versus -host disease (c-GVHD) resembles the appearance of fibrosis in scleroderma (SSc). Recent studies identified distinctive structural changes in the superficial dermal microvasculature and matrix of SSc skin. We compared the dermal microvasculature in human c-GVHD to SSc to determine if c-GVHD is a suitable model for SSc.

**Methodology/Principal Findings:**

We analyzed skin biopsies of normal controls (n = 24), patients with SSc (n = 30) and c-GVHD with dermal fibrosis (n = 133)). Immunostaining was employed to identify vessels, vascular smooth muscle, dermal matrix, and cell proliferation. C-GVHD and SSc had similar dermal matrix composition and vascular smooth muscle pathology, including intimal hyperplasia. SSc, however, differed significantly from c-GVHD in three ways. First, there were significantly fewer (p = 0.00001) average vessels in SSc biopsies (9.8) when compared with c-GVHD (16.5). Second, in SSc, endothelial markers were decreased significantly (19/19 and 12/14 for VE cadherin and vWF (p = <0.0001 and <0.05), respectively). In contrast, 0/13 c-GVHD biopsies showed loss of staining with canonical endothelial markers. Third, c-GVHD contained areas of microvascular endothelial proliferation not present in the SSc biopsies.

**Conclusions/Significance:**

The sclerosis associated with c-GVHD appears to resemble wound healing. Focal capillary proliferation occurs in early c-GVHD. In contrast, loss of canonical endothelial markers and dermal capillaries is seen in SSc, but not in c-GVHD. The loss of VE cadherin in SSc, in particular, may be related to microvascular rarefaction because VE cadherin is necessary for angiogenesis. C-GVHD is a suitable model for studying dermal fibrosis but may not be applicable for studying the microvascular alterations characteristic of SSc.

## Introduction

After allogeneic hematopoietic cell transplantation (HCT), 40–60% of recipients who survive at least 6 months after HCT will develop chronic graft-vs.-host disease (c-GVHD) [1]. C-GVHD is a complex multisystem syndrome with overlapping features of immunodeficiency and several of the naturally occurring autoimmune disorders. A prominent clinical feature of c-GVHD is a debilitating fibrosing skin disease whose gross and histologic features resemble both scleroderma (SSc) and, less commonly, morphea [2], [3]. Because of these similarities, a number of murine models of c-GVHD [4], [5] have been used to study the potential mechanisms underlying SSc. These murine models of c-GVHD that have produced dermal fibrosis have not successfully recapitulated the characteristic vascular abnormalities in SSc including intimal hyperplasia, rarefaction of vessels and pulmonary hypertension [5]. Similarly, other non-GVHD models including the tight skin mouse and bleomycin induce fibrosis but do not clearly produce the changes seen in the vasculature of SSc [6]–[8].

Data regarding the vascular pathology associated with cutaneous c-GVHD are limited. Biedermann [9] et al. investigated the relationship of superficial dermal microvessels in the papillary dermis of patients with acute GVHD (a-GVHD) and c-GVHD for signs of vascular injury and dermal fibrosis. Utilizing staining with *Ulex europaeus* agglutinin, they described a loss of superficial dermal microvessels (a-GVHD less than c-GVHD) accompanied by perivascular infiltration of activated (GMP17+) CD8+ CD8+ T cells in skin. Based on these observations, Biedermann et al. hypothesized that the endothelial cells of the recipient's superficial dermal microvessels in cutaneous c-GVHD are targeted by the alloreactive donor cytotoxic T-lymphocytes leading to blood vessel loss, impaired blood perfusion, hypoxia, and tissue fibrosis. Other studies of human intestinal GVHD have emphasized the damaged capillary bed [10], [11], defined as either the presence of apoptotic cells associated with capillaries, or the presence of thrombotic microangiopathy.

In our previous investigation of scleroderma (SSc) skin biopsies, we performed immunohistologic staining with antibodies for canonical endothelial markers, CD31 (platelet endothelial cell adhesion molecule), vWF, and VE cadherin. As early as 3 months after the initial diagnosis of SSc, we found rarefaction of small vessels [12] and loss of these markers. Because VE cadherin is essential for tube formation in developing blood vessels [13], we described the loss of microvessels with the loss of these markers in the remaining cells as an “anti-angiogenic” phenotype.

In the present study, we compared skin biopsies from patients with cutaneous fibrotic c-GVHD, SSc, and normal controls. We evaluated changes characteristic of the vasculopathy of scleroderma including diffuse intimal hyperplasia, endothelial phenotype and capillary rarefaction [14], [15]. Dermal matrix characteristics were evaluated with hyaluronan staining and graded by a dermal fibrosis scoring system (DFS) which we have used in a previous publication [16].

The results showed that c-GVHD lacks some of the distinctive changes we have previously described in scleroderma.

## Results

### Dermal fibrosis and other histological features of skin in SSC and c-GVHD

#### C-GVHD

The methods used to score DFS are shown in Table 1. C-GVHD: The clinical and histologic features of the skin from 13 biopsies with c-GVHD are described in Table 2. Skin biopsies 1 and 2 in table 2 were on different dates and locations from the same patient. All 12 patients satisfied the NIH clinical and histologic consensus criteria for c-GVHD [1], [3]. Skin biopsies were obtained at a median of 642 (range, 320–1323) days after HCT. Nine of the 13 biopsies had fibrosis with grade 3 or higher DFS score. The fibrosis involved 50–75% of the dermis, and the average DFS score was 3.15 for the c-GVHD biopsies. Four of the biopsies had morpheic clinical and histologic features. The c-GVHD group displayed the histologic spectrum of c-GVHD from a lichen planus -like lesions (lichenoid in the NIH classification) with epidermal acanthosis, hyperkeratosis and hypergranulosis, intense inflammation along the dermal epidermal junction with limited fibrosis, to diffuse end-stage fibrotic disease with DFS 4 and 5. With the exception of c-GVHD skin biopsy 2 with morphea-like disease involving the deep dermis, all c-GVHD biopsies had fibrotic disease involving at least the upper dermis. Earlier stages of c- GVHD with lichenoid appearance had prominent apoptosis along the basilar layers of the epidermis and follicular and dermal appendages.

**Table 1:** Dermal Fibrosis scoring*.

0	No homogenization but there may be atrophic thin straightened colleen bundles with increased amounts of interstitial ground substance
1	Less than 25% sclerosis with residual foci; some residual straightening or eosinophilic collagen bundles may be present
2	Focal sclerosis – less than 50% overall
3	Incomplete homogenization with spaces between the collagen bundles with 50% to 75% sclerotic change
4	Pandermal sclerosis without obvious expansion of the lower reticular dermis with some sparing of the perieccrine adipose tissue
5	Pandermal sclerosis with homogenization from the papillary to the reticular dermis; includes obvious widening of the reticular dermisbelow the eccrine coils with extension into the hypodermis and formation of fibrous septa

* DFS was based on H&E stained collagen and dermal matrix changes Blood 2007:110,1388.

**Table 2:** Clinical and Histologic Findings in 13 Patients with Chronic GVHD.

Code	Days post tranplant	DFS Score	Clinical Findings	Histology Comment
GVHD 1	593	**5**	Morphea	pandermal sclerosis, scapular lesion
GVHD 2	623	**3**	Morphea	morphea with deep sclerosis in lower 66% of dermis and lichenoid activity but without apparent subepidermal sclerosis
GVHD 3	382	3	Tight skin hair loss oral and liver involvement	Dense sclerosis of upper 33% dermis and sclerosis in deep retic d.
GVHD 4	810	1	Tight skin with limited range of motion	lichenoid patchy subepid sclerosis, marked vascular prolif & inflam
GVHD 5	991	5	Sclerosis with limited range of motion oral and ocular involvement	pandermal sclerosis
GVHD 6	642	5	Extensive scleroderma oral and ocular involvement	dense pan dermal sclerosis
GVHD 7	320	1	Extensive lichen planus-like lesions without sclerosis	lichenoid, mild subepid sclerosis, perivascular inflamation
GVHD 8	618	5	Morphea	pan sclerosis
GVHD 9	1323	0–1*	Dyspigmentation, tightness, lichenoid lesions, hair loss	lichenoid, focal subepidermal sclerosis
GVHD 10	1142	3	Prior fasciitis, dyspigmentation, inactive when biopsied	sclerotic with some residual nl dermis in mid-reticular zone
GVHD 11	437	1	Oral and ocular sicca, skin tightness with rash and deep sclerosis	lichenoid, thin band of subepid dermal sclerosis
GVHD 12	1076	4	Lichenoid plaques, fibrosis of the hands	pan dermal sclerosis, ectatic superficial venules
GVHD 13	985	3	Left flank morphea, poikiloderma with sclerosis	dense deep reticular dermal and fascial sclerosis. Subepidermal sclerosis with sparing of upper reticular derma

#### SSc

All SSc patients had clinical evidence of diffuse cutaneous skin fibrosis. Biopsies were primarily from areas of severe dermal involvement such as the forearm. In contrast to the skin biopsies from the c-GVHD group, the SSc biopsies had limited inflammation and little or no epidermal apoptosis. The extent of fibrosis was histologically comparable to the c-GVHD biopsies, and the SSc skin biopsies scored a median DFS of 3. Biopsies from c-GVHD patients with severe fibrotic disease were indistinguishable histologically from biopsies of patients with untreated and severe SSc.

### Fibrotic changes in SSc and c-GVHD

**Figure 1:**
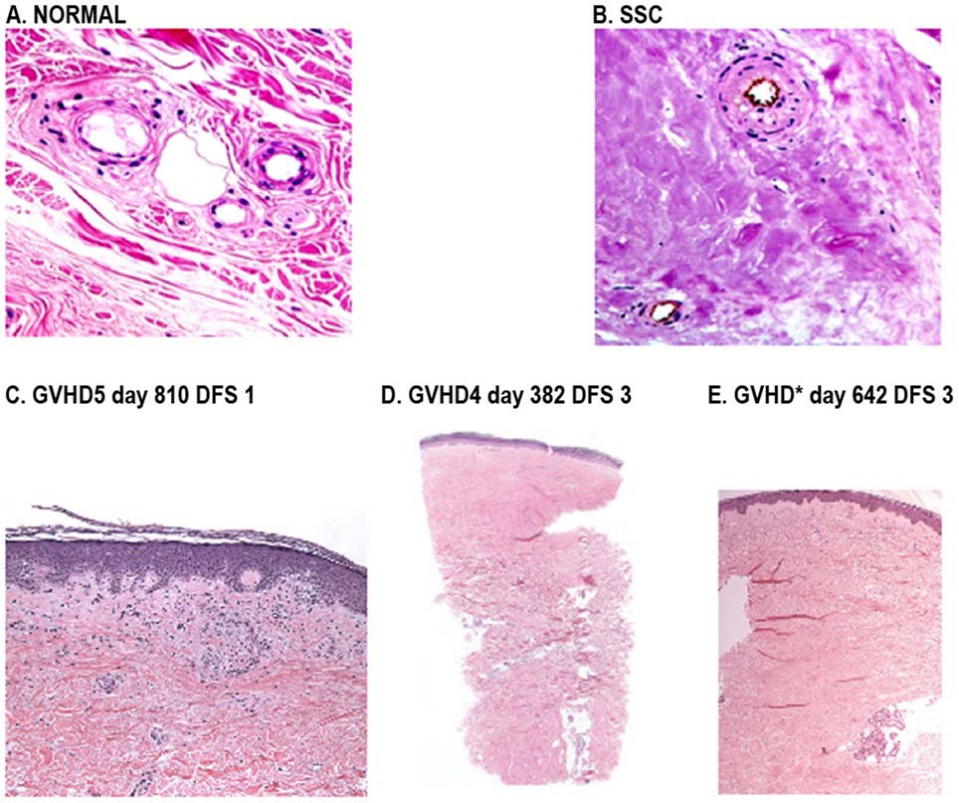
Dermal fibrosis in skin biopsies of Normal, c-GVHD and SSc skin. Normal skin stained A. with H&E has collagen bundles with curlicue pattern and a dermal fibrosis score (DFS) of zero. 20× magnification B. SSc depicting a DFS of 5 at 20× magnification. C. GVHD day 810 DFS 1: epidermis has patchy lichenoid lymphocytic inflammation along the dermal epidermal junction the widened and papillary dermis contains loosely scattered myofibroblasts and clusters of small proliferated vessels (lower power is 10×) D. GVHD4 day 382 DFS 3: the entire papillary and upper portion of the reticular dermis are replaced by dense fibrosis in a the top-down direction E. GVHD8 day 642 DFS 5: Fibrotic disease throughout the dermis with replacement of the loose papillary dermal collagen and reticular dermal collagen bundles by dense smudgy collagen.

Compared with normal skin (Figure 1A), the dermal collagen bundles were replaced by a dense waxy fibrotic matrix in both SSc (Figure 1B) and c-GVHD (Figure 1 C, D and E). The progression of the dermal fibrosis in patients with c-GVHD proceeded from the epidermis down [17]. In contrast, the skin biopsies from some of the SSc cohort had relative preservation of the dermal collagen bundles in the upper half of the dermis, while the lower dermis was intensely fibrotic.

There was increased staining for hyaluronan within the epidermis and dermis of biopsies from both SSc and c-GVHD. The elevated level of hyaluronan was especially concentrated around blood vessels and contrasted with the minimal or absent staining in the normal control biopsies (shown in supplemental Figure S1). The hyaluronan stain in the epidermis was also markedly increased in both fibrotic diseases compared with normal control biopsies (data not shown).

### Intimal hyperplasia in SSc and c-GVHD

**Figure 2:**
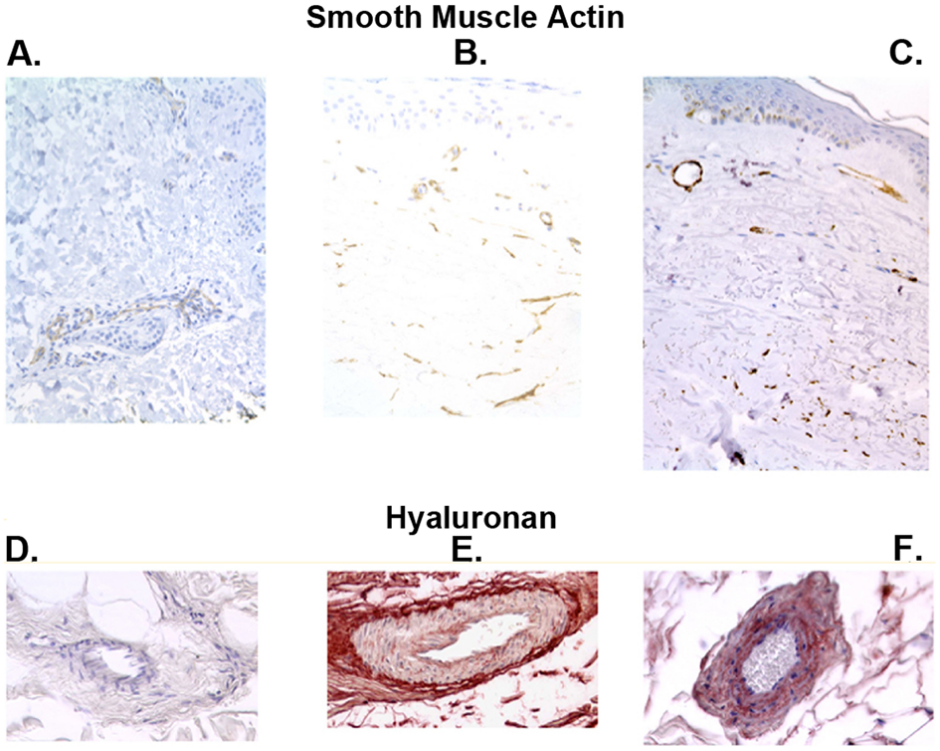
Myofibroblasts and intimal hyperplasia in Normal, GVHD and SSc skin. Smooth muscle markers were used to depict mural cells and pericytes. A. Smooth muscle actin (SMA) in normal control with normal positive cells in vessels. B. C-GVHD biopsy with many SMA+ myofibroblasts C. Representative SSc biopsy with SMA+ myofibroblasts in dermis slightly lower than c-GVHD Hyaluronan can be used to show abnormally thickened vessels since this marker tends to accumulate on intima and in smooth muscle cells after injury. D. Normal skin showing unaltered vessels in lower dermis. E. C-GVHD biopsy showing thickened vessels with some hyaluronan in the vessel wall. F. Similarly thickened vessels in SSc with increased hyaluronan.

The arterial microvessels of SSc displayed mural thickening including medial smooth muscle hyperplasia and intimal cell hyperplasia. Hyaluronan staining identified abnormally thickened vessels since this marker accumulates in intima formed after injury [18], (Figure 2 A–C). Intimal hyperplasia was present in small and large vessels of both SSc and c-GVHD and was absent from normal control biopsies. The elastin layer delimiting the border between media and intima was often obscured in SSc and the vessels appeared thickened and abnormal. We stained the biopsies for smooth muscle actin (SMA) and smooth muscle myosin (SMMHC) and some of these photomicrographs can be seen in supplemental Figure S2. We found that there were many more layers of smooth muscle cells in the arterial walls of the SSc and c-GHVD skin compared with normal controls (Table 3). We could not find a difference in the arterial smooth muscle thickness between c-GVHD and SSc (Table 3). An increase of dermal myofibroblastic cells were identified by immunostains for the smooth muscle markers in roughly 85–70% of the biopsies with c-GVHD and SSc, respectively (Figure 2 E and F). There was no difference in myofibroblast numbers between SSc and c-GVHD, however, both diseases were significantly different from normal controls (Table 3).

**Table 3:** Quantification of Immunohistochemistry scores.

Category	Normal	SSc	cGVHD	NL vs. SSc	NL vs. cGVHD	SSc vs. cGVHD
Average DFS 0–5	0.1	2.7	3.1	<0.05b	<0.05b	nsb
SMA myofibroblasts #positive/#total	0/16	8/11	5/7	<0.0001a	<0.05a	nsa
SMMHC intimal hyperplasia #positive/#total	0/7	10/11	5/6	<0.05a	<0.05a	nsa
SMMHC mypfibroblasts #positive/#total	0/7	8/11	5/6	<0.05a	<0.05a	nsa
Loss of VE Cadherin #positive/#total	0/15	19/19	0/13	<0.0001a	nsa	<0.0001a
Loss of vWF #positive/#total	0/8	12/14	0/13	<0.05a	nsa	<0.05a

ns–not significant.

a–Fisher's exact test.

b-Mann Whitney test.

### Capillaries are rarefied (reduced) in SSc but not c-GVHD

**Figure 3:**
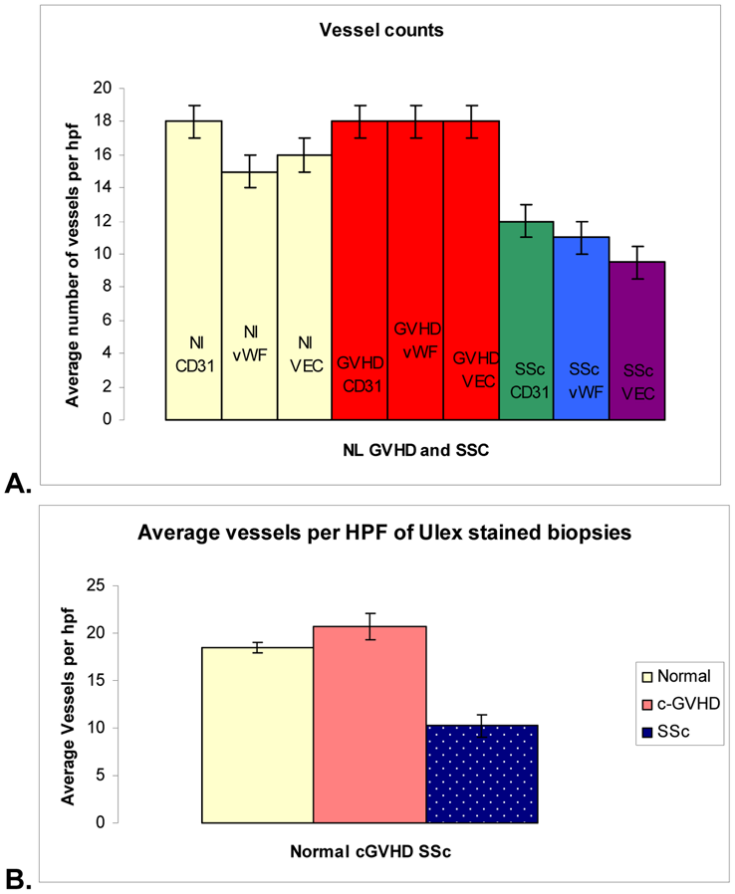
Capillary counts in c-GVHD compared with normal controls and SSc. Vessel counts by antibody staining A. show SSc has significantly fewer vessels than both other patient groups. Yellow bars show normal tissue vessel counts, red bars show c-GVHD vessels counts, and green blue and purple bars show SSc biopsies stained with CD31, vWF and VE Cadherin respectively. Normal controls and c-GVHD counts are not significantly different from each other regardless of marker used. These counts are also not significantly different within the group (i.e. CD31 stained normal tissue has similar number of vessels to normal tissue stained with vWF). SSc has fewer vessels than c-GVHD and normal tissue with every antibody used to label endothelial cells. In addition, the remaining vessels in SSc (total represented by green bar) a significant proportion of these vessels have lost expression for vWF (blue bar) and VE Cadherin (purple bar). B. Graph shows the results of vascular density quantification after Ulex lectin staining. Normal controls and c-GVHD biopsies are represented by the yellow and orange bars, and are not significantly different. Blue bar represents the average number of vessels in SSc, and is significantly less than the other two groups.

The results of superficial dermal microvascular vessel density/hpf are displayed graphically in Figure 3A by triplet sets of bars representing the quantitations using method 1 as defined in the materials and methods. Endothelial cells were identified by antibody staining with CD31, vWF, and VE cadherin. The yellow colored bars in Figure 3A represent normal controls, the red c-GVHD, and the multicolored SSc. The error bars represent one standard error. Average number of vessels for normal skin stained with CD31 is 17.9±3 for vWF 14.6±3 and for VE cadherin 16.2±3. The average number of vessels in c-GVHD biopsies stained with CD31, vWF and VE cadherin was 17.8±3, 18.1±3, and 18.3±4, respectively. The values for each antibody determination were not significantly different.

The lowest vessel density with each of the three antibodies occurred in the SSc biopsies. The bar representing average vessels/hpf was; green CD31 12.2±2, blue vWF 10.8±2, purple VE 9.5±2. As we reported in a previous study [12], SSc biopsies showed decreased expression of vWF and VE cadherin when compared with CD31 (see table 3). Both normal controls and c-GVHD had significantly more vessels than SSc biopsies when stained with CD31 (p<0.00001 and p<0.05, respectively) but there was no difference between c-GVHD and normal biopsies. Normal controls and c-GVHD tissue stained with antibodies for vWF had significantly more vessels than SSc biopsies stained with the same antibody (p<0.05 for both). Again there was no difference when c-GVHD was compared to normal controls. Comparing the VE cadherin staining in the three populations, normal controls or c-GVHD biopsies had significantly higher vessels/hpf than SSc (p<0.05). VE cadherin staining of normal versus c-GVHD showed no significant difference. C-GVHD had the same or slightly more vessels than normal tissue.

Since Biedermann's paper reported that c-GVHD had fewer vessels than normal controls using [9], we repeated our vessel density analysis using *Ulex* as in their study. We compared 13 c-GVHD biopsies to 10 normal and 12 SSc biopsies (Figure 3B) In contrast to the results from Biedermann [9], c-GVHD biopsies had an average of 20.75 vessels/hpf, slightly increased from normal controls that had 18.45 vessels/hpf (p = 0.12). The SSc biopsies stained with *Ulex* differed significantly from both c-GVHD and normal controls, with an average number of 10.25 vessels per hpf (p<0.00001 for both). In sum, we found that using *Ulex* staining the vessel density/hpf in c-GVHD was not decreased, was approximately the same as the normal controls, and the counts were very similar to those found using the other three antibodies.

### Endothelial markers are lost in SSc but not c- GVHD

**Figure 4:**
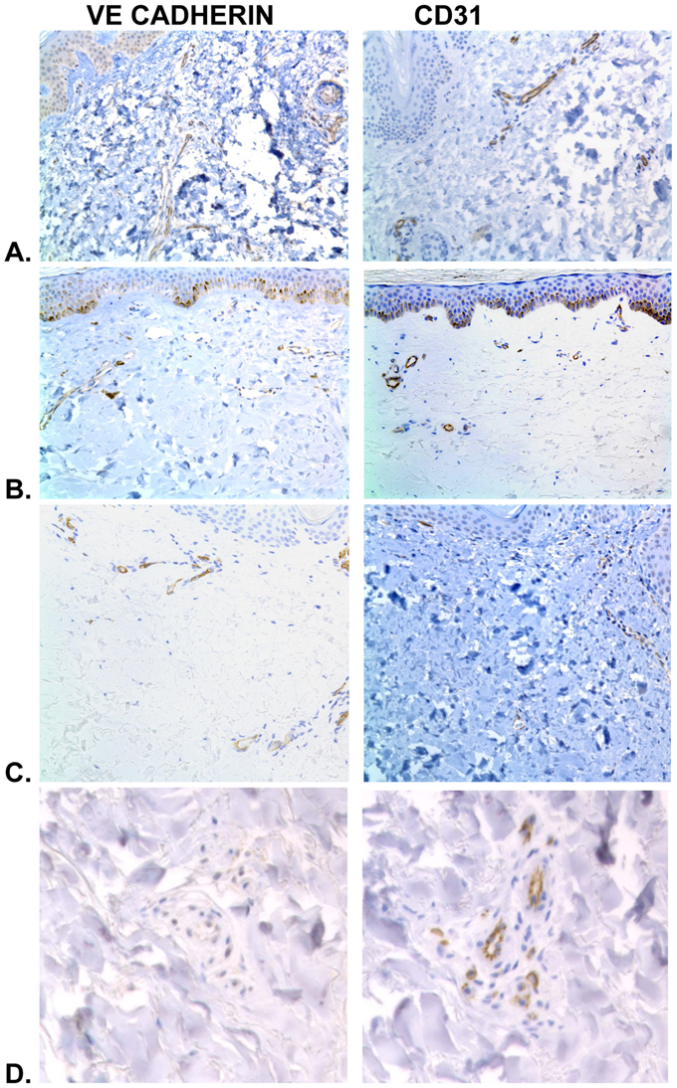
Only SSc has lost endothelial cell markers. Depicted in A. Normal skin stained with VE cadherin and CD31 showing similar patterns of staining. The same patterns are seen in B. early c-GVHD stained with the same two antibodies and C. late c-GVHD similarly stained with VE cadherin and CD31. The only skin samples which showed loss of VE cadherin were the SSc biopsies D.

SSc biopsies had a significant reduction of vessels/hpf stained by vWF and VE cadherin compared with the same biopsies stained with CD31 (Table 3). Beyond rarefaction, however, our previous study of SSc reported changes in the phenotype of the endothelium in the remaining vessels. We confirmed the lack of vWF and VE cadherin staining in the endothelium of biopsies by using method 3 (defined in materials and methods) comparing the photomicrographs of serial section for vessels which were positive for CD31 but negative for either of the other two antibodies. Only SSc had vessels that were clearly positive for CD31 while negative for the other canonical markers (Table 1). Representative photomicrographs show the endothelial markers in normal skin and c-GVHD skin are quite visible (Figure 4 A-C) whereas VE cadherin is missing from vessels in SSc skin (Figure 4 D). Similarly *Ulex europaeus* lectin was seen in every vessel stained with CD31 when the biopsies are compared side by side (data not shown). As we have previously published, SSc has not only lost vessels, the remaining vessels are only sometimes positive for *Ulex*.

### Focal areas of capillary proliferation with increased endothelial markers are identified c- GVHD

**Figure 5:**
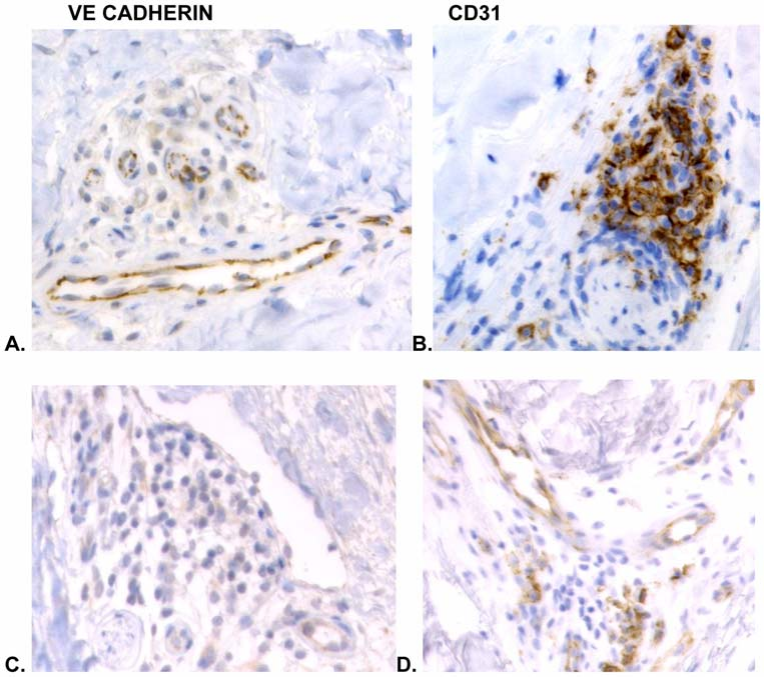
Endothelial markers in clumps of microvascular proliferative formations in GVHD but not SSc. C-GVHD with areas of microvascular proliferation A. Many VE cadherin positive cells present throughout the structure. Some of the endothelial cells within the structure have a normal aggregated stain pattern with VE cadherin at the junctions, wheras in proliferating and migrating endothelial cells the VE cadherin is spread out in the cytoplasm. VE cadherin spread in the cytoplasm is a typical finding in proliferating endothelial cells in glomeruloid bodies in skin. B. CD31 stained biopsies of c-GVHD with glomeruloid bodies with many cells positive for CD31 appearing in large clumps. Similar appearing structures in SSc did not have endothelial markers present in the cells. C. SSc, VE cadherin has few weakly positive cells lining the lumens and very few readily identifiable proliferating endothelial cells D. SSc,CD31 positive cells are sparse in these areas structurally resembling microvascular proliferation. Although multiple lumens are present there are very few CD31 positive clumps of cells.

The term lichenoid defined by the NIH consensus criteria for c-GVHD refers to the early cutaneous histologic changes where there is epidermal orthokeratosis, hypergranulosis, acanthosis, and extensive inflammation with apoptosis along the basal layer with damage to the rete ridges [3]. In the course of our vessel studies we noted that some c-GVHD biopsies, especially those with a lichenoid histologic picture, had areas of microvascular proliferation in the papillary dermis that were not present in normal controls. The structure of these clusters of cells resembled glomeruloid bodies [19]. The cells in these structures were positive for endothelial markers (Figure 5 A and B). A few SSc biopsies had similar structures, however, the endothelial cell markers were sparse within them (Figure 5 C and D). In both SSc and c-GVHD there were cells in these structures positive for Ki67 implying that glomeruloid bodies represent proliferative structures. Additional photos of the proliferative areas in c-GVHD and SSc are available in supplemental Figure S3.

## Discussion

In spite of similarities in cutaneous fibrosis, the clinical presentation of c-GVHD differs from SSc in several ways including the absence of Raynaud's phenomenon, a general lack of the ischemic changes in distal extremities, characteristic autoantibody profile of SSc and internal organ disease as is seen in diffuse SSc. The observations in this study moreover, show that the superficial dermal microvessels in c-GVHD differ from those seen in SSc by having neither capillary rarefaction nor the loss of endothelial cell specific markers we described before [12]. In fact, many biopsies of c-GVHD were associated with an increase of small vessels and in some we found small plexiform microvascular proliferations resembling glomeruloid bodies (as seen in Fig. 4). These structures resemble capillary malformations resulting from local overexpression of VEGF [20], [21] suggesting that VEGF may be overexpressed in the skin of c-GVHD patients as it is in the skin of patients with SSc [22]. This hypothesis is supported by Shimura et al who reported an increase in circulating levels of VEGF in c-GVHD [20]. Only one end stage pansclerotic c-GVHD biopsy showed vascular rarefaction.

Our results disagree with the findings reported by Biedermann et al. [9]. They claimed loss of capillaries resulted from targeting of host endothelial cells by alloreactive donor cytotoxic T-cells in c-GVHD results in loss of the capillaries with subsequent ischemia and ischemia driven dermal fibrosis. Their hypothesis is consistent with in vitro studies showing that allogeneic T cells can kill endothelial cells [23]. Shimura et al. [24] provided support for Biedermann's hypothesis. In five patients with sclerodermatous c-GVHD, Shimura et al. found a reduction in circulating CD34+/CD133+ putative endothelial precursors when compared to controls and c-GVHD patients without fibrosis of the skin. Biedermann et al. inferred endothelial injury by the histologic appearance of reactive swollen endothelium and the presence of elevated vWF multimers. Personal experience (HMS) in evaluating thousands of c-GVHD biopsies as well as observations by Biedermann et al. over a wide range of times after allotransplantation found no evidence of endothelial injury in the form of hemorrhage, edema, thrombosis, or endothelial apoptosis.

The disparity between our conclusions with *Ulex* and the data from Biedermann et al. may be explained by three differences in the studies. First, we do not regard elevation of vWF multimers as evidence of an alloreactive endothelial injury but rather as an indication of perturbation of the endothelium involved in the inflammatory process by the homing of alloimmune lymphocytes and the surrounding cytokine milieu [25], [26]. Additionally vWF is elevated in response to some kinds of drug therapy [27] and is considered to be a non-specific acute phase reactant in the inflammatory process [28].

The second difference is the criteria for defining endothelial cells. *Ulex* lectin detects glycosylation products of blood group antigens rather than a protein or RNA expressed by the endothelial cell. The *Ulex* antigen is a specific fucose moiety generated by the action of the fucosylase FUT1 [29]. Published reports show that fucosylases can compete with each other and with other transferases especially in the presence of inflammation [30]. In contrast CD31 is the marker most commonly used to identify vascular endothelia and VE cadherin is made uniquely by endothelial cells [31]. The third difference is the morphometric method used to count vessels. In our study of SSc we saw a specific loss of small thin walled vessels (dermal capillaries) [12]. Biedermann et al. describe measuring the total surface area stained by *Ulex* lectin without regard to vessel size. An examination of Figure 2 suggests that they may have seen a shift in mean vessel size rather than numbers. A decrease in total vessel circumference may simply mean that the vessels are smaller in c-GVHD, which is consistent with increased numbers of small newly made capillaries.

Although we did not see capillary rarefaction in c-GVHD we did see intimal hyperplasia, another important vascular pathology. Both SSc and cGVHD have changes in smooth muscle thickness in vasculature of the skin. Diffuse intimal hyperplasia similar to that seen in SSc and c-GVHD is solid organ transplant of heart, lung, and kidney. Fibrosis accompanies the vascular changes of transplant arteriosclerosis [32].

Despite the similarities between SSc and c-GVHD there is a difference in the pattern of fibrotic change. With the exception of one patient with morphea-like changes in only the lower dermis, c-GVHD biopsies showed dermal fibrosis proceeding in a top-down manner, spreading downward from the papillary and adnexal dermis surrounding destroyed follicles [3]. This is opposite from the usual pattern in SSc where fibrosis extends from the hypodermis upward.

The cell usually implicated in fibrosis of SSc is the myofibroblast, defined as a fibroblast with abundant expression of smooth muscle contractile genes [33]. Both SSc and c-GVHD have spindle-shaped, smooth muscle actin positive myofibroblasts scattered within the increased matrix.

The molecular stimuli for fibrosis in SSc and c-GVHD may be similar. Fibrosis is driven by soluble mediators from differentiated type 2 immune response [34] including Il-4, Il-13 and TGF- β[35], [36]. Experimental data indicate that TGF-β, a potent driver of fibrosis is involved in both the genesis of c-GVHD [5] and SSc [37]. In vitro data suggest that myofibroblast formation is driven by PDGF and TGF β [38], [39]. Experimental animal models enhancing TGF β activation (e.g. by mutations in the TGF β binding domain of fibrillin) demonstrate severe fibrotic reactions [40]. Recent studies in mice show the fibrosis is antagonized by losartan[41]. Svegliati [42], [43] has described an autoantibody in patients with SSc and c-GVHD which reacts with the PDGF receptor to turn on fibrosis in vitro. Two clinical trials are underway that may test the therapeutic implications of this hypothesis. First, rituxan, an anti CD20 with reactivity against B cell activity, has been used with some success in treating obliterative bronchiolitis and skin manifestations of c-GVHD [44]. Second, a multi-institutional study with imantinib, a tyrosine kinase inhibitor is directed at shutting down activation of fibroblasts by blocking PDGF signaling.

In summary, while c-GVHD and SSc may share common fibrotic mechanisms, our data suggest that rarefaction and loss of endothelial-specific markers may be specific to SSc and offer a clinical target in its own right. Recent clinical trials using autologous HCT for severe SSc showed arrest or reversal of dermal fibrosis associated with regeneration of dermal capillaries and restitution of the endothelial phenotype [12], [16]. Clinical trials of allogeneic grafts into SSc patients are ongoing, and a percentage of these patients these are expected to develop c-GVHD. Presence or absence of VE cadherin and vascular density may have utility in these studies to distinguish c-GVHD from persistent SSc in allogeneic stem cell transplant recipients. The mechanism for the regenerative effect of HCT is not yet known, however, it is intriguing to consider the possibility that the critical result is endothelial regeneration.

## Materials and Methods

### Ethics Statement

The investigations in this study were based on skin biopsies supplied in collaboration and analyzed according to the University of Washington Institution Review Board approved protocols for human studies (#29426 and #D3-8439-D 03). The biopsies were provided by Stanford University, University of California San Francisco, Boston University, Fred Hutchinson Cancer Research Center, Seattle Cancer Care Alliance and the University of Washington Medical Center. Biopsies were selected from stored tissue that was collected for other studies, de-identified prior to delivery to our institution for analysis, and written consent was unnecessary for the specific purpose of this study.

### Patients

The biopsies came from 24 normal controls, 30 SSc patients and 12 c-GVHD patients with patient 1 having two biopsies done on separate days. Additional details on the normal and SSc patients are provided on supplemental tables S1 and S2. Clinical data was available for 22 of 24 normal controls ages range from 36 to 73 with the majority of biopsies from forearm although there were also samples from scalp and torso. Clinical data was available for all SSc patients, ages range from 32 to 71 and disease duration range from 6months to 6 years. Most of the SSc biopsies were from the forearm, although there were also samples from torso and thigh. The skin biopsies with c-GVHD were obtained from the University of Washington affiliated institutions.

### Dermal fibrosis score

After reviewing the pathology archive between 2005–2007, we identified skin biopsies from 13 patients whose biopsies had subtotal to complete dermal sclerosis and satisfied the NIH histopathology requirements for c-GVHD [3]. Table 2 shows the age, day post-transplant and their dermal fibrosis score (DFS) [45] which reflects the percentage of sclerosis in the full thickness of dermis with by quintiles; grade 1<25%, grade 2 25–50%, grade 50–75%, grade 4 pandermal sclerosis, grade 5 pandermal sclerosis with extension into the hypodermis The evaluation of DFS was done on sections stained with H&E.

### Immunohistochemistry (IHC)

In order to comprehensively define the components of the small vessels the antibodies used were directed at three general categories; first, endothelium, physiologic antibodies CD 31, VE cadherin, vWF and the lectin *Ulex europeus* which stains fucosylated substances on the endothelium and basement membrane,), second, vascular smooth muscle (non muscle myosin heavy chain, smooth muscle myosin heavy chain and smooth muscle actin) and third, intimal matrix (hyaluronan). Evidence of active cell cycle proliferation was done with Ki67. The manufacturer, clone, dilution, and pretreatment details for each primary antibody are summarized in supplemental Table S3. Immunohistochemistry for VE cadherin, vWF, smooth muscle myosin heavy chain (SMMHC) and some α smooth muscle actin (SMA) was performed at Phenopath Laboratories (Seattle, Washington). Some of the immunostaining for CD31, some α smooth muscle actin, and some vWF was performed at the Seattle cancer care alliance pathology laboratory using similar protocols.

Immunohistochemistry for hyaluronan was done at the Wight lab: Paraffin sections were dewaxed, endogenous peroxidases were blocked using H_2_O_2_ in methanol, and rehydrated in a series of graded ethanol. For hyaluronan affinity histochemistry, rehydrated tissue sections were blocked in 1%BSA-PBS and incubated with biotinylated hyaluronan binding peptides (4 ug/ml) and hyaluronidase treatment of sections was used as a negative control. The slides were incubated for 30 minutes with the ABC reagent supplied in Vector Elite Universal Kit (Vector Labs #PK6100). Detection was performed with Vector NovaRed substrate to create a red color (Vector Labs # SK-4800), the slides were then counterstained with Gills #3 Hematoxylin, dehydrated in ethanol series, cleared in xylene and mounted with Quik-Mount (Research Products International Corp., Mount Prospect, IL).

### IHC Scoring methodology

Quantitative microscopy was done using an Nikon E400 Microscope with a 20x optic, numerical aperture **0.75** with **10×** eye pieces.

#### Myofibroblasts score (SMA, SMMHC)

Non-perivascular cells identified as existing isolated in the matrix (i.e.) not part of a smooth muscle fiber. Biopsies were considered positive if there were groups (more than 3) of these scattered cells present. Biopsies were scanned and presence or absence recorded.

#### Intimal/Smooth Muscle Vascular Pathology (SMA, SMMHC)

The biopsies were scanned until positive vessels were located or the entire biopsy had been examined for blood vessels with greater than 3 layers of smooth muscle in the blood vessel wall. The range and average number of normal skin vessels with greater than 3 layers per height per hpf was determined. The mean was calculated and assuming a normal distribution, biopsies within two standard deviations of the positive range were scored as positive. Biopsies with an average number of cells lower than the cutoff were scored as negative. Negative and positive were recorded in 2×2 frequency tables.

For our purposes, superficial dermal vessels were defined as those vessels within one hpf of the epidermis. All the vessels (clumps of cells or cells surrounding a lumen that were positive for the endothelial marker) within one hpf of the epidermis were counted in four separate sections of the biopsies. This method was designed to include capillaries and sections of tissue that don't include the lumen of the vessel in the cell counts.

#### Vessel quantification

The quantitative vascular counts were performed by comparing the four endothelial immunohistologic stains on serial sections. The quantitation focused on the papillary dermis located just below the epidermis, since the majority of capillaries from skin are found in this part of the dermis. Four contiguous high power fields (hpf) or the entire breadth of the biopsy was counted. The comparative photomicrographs of serial sections with different immunohistologic stains were taken of the upper dermis extending from just below the epidermis. All quantitations were done on coded section by at least one blinded observer. If the vascular quantitation counts from a given biopsy differed substantially between endothelial stains, the quantitation was repeated by at least two additional blinded observers to verify the significant differences using different vascular antibody stains.

### Determination of vessel rarefaction

The average vessel counts with the four endothelial marker antibodies for normal control biopsies were compared to those counts from c-GVHD and SSc biopsies. Differences expressed using students t-test statistics are shown in Figure 3.

### Loss of endothelial phenotypic markers vWF and VE cadherin

Loss of vascular staining vWF and VE cadherin was determined by comparison with the CD31 counts using students t test statistics (Figure 2.) The loss of endothelial cell VE cadherin and/or vWF immunoreactivity in SSc biopsies was verified was by comparing photographs of serial sections stained with VE cadherin and vWF to the same vessel which displayed immunoreactivity to CD 31. Positive (for loss) or negative (no loss of staining) was recorded in 2X2 table and statistically analyzed by fishers exact significance test. Results are shown in Table 1.

#### Statistics

The stain scores for intimal hyperplasia, vessel quantification method 3, smooth muscle capillary scores and myofibroblast scores were recorded as frequency data and were analyzed for significance using Fishers Exact Significance test or the Mann Whitney test. Frequency data were obtained according to above scoring systems and 2×2 tables were used to record differences between groups. P values were calculated with one tailed probability value. The numbers per hpf for the remainder of the stains and methods 1 and 2 for vessel quantification were recorded and continuous variables and therefore tested for significance with the students t test. Two tailed p values were used to show significance.

## Supporting Information

Figure S1 Histochemistry of scleroderma dyregulation of matix molecules A. Increased hyaluronan is present in Ssc and GVHD in the A dermal matrix and in B. areas of microvascular proliferation/cellularity.
(1.72 MB TIF)

Figure S2 Intimal hyperplasia or smooth muscle hyperplasia in SSc and c-GVHD A. lichenoid c-GVHD SMMHC antibody depicts multiple layers of smooth muscle in skin vessels. No such vessels were found in any of the many full thickness biopsies of normal controls skin. B. Sclerotic c-GVHD stained with SMA, shows both larger and smaller vessels with multiple layers of smooth muscle cells, with scattered myofibroblasts and increased cellularity highlighting the inflammatory nature of c-GVHD C. SSC stained with an antibody to SMMHC shows thickened vessel walls around swollen endothelial layer
(1.80 MB TIF)

Figure S3 Endothelial markers in clumps of microvascular proliferative formations in GVHD Sclerotic cGVHD with areas of microvascular proliferation as defined by A. vWF B. SMA similar formations were seen in lichenoid cGVHD as defined by E. vWF and F. SMA similar appearing structures in SSc did not have endothelial markers present in the cells G. VE cadherin is shown with H. CD31, positive cells are sparse in these areas, although multiple lumens are present
(2.56 MB TIF)

Table S1 (0.07 MB DOC)

Table S2 (0.06 MB DOC)

Table S3 (0.04 MB DOC)
