# Supplementary figures and images for: Cutaneous Chronic Graft-Versus-Host Disease Does Not Have the Abnormal Endothelial Phenotype or Vascular Rarefaction Characteristic of Systemic Sclerosis
Source: PLoS One. 2009 Jul 9;4(7):e6203. doi: 10.1371/journal.pone.0006203 (PMC2705674; doi:10.1371/journal.pone.0006203)

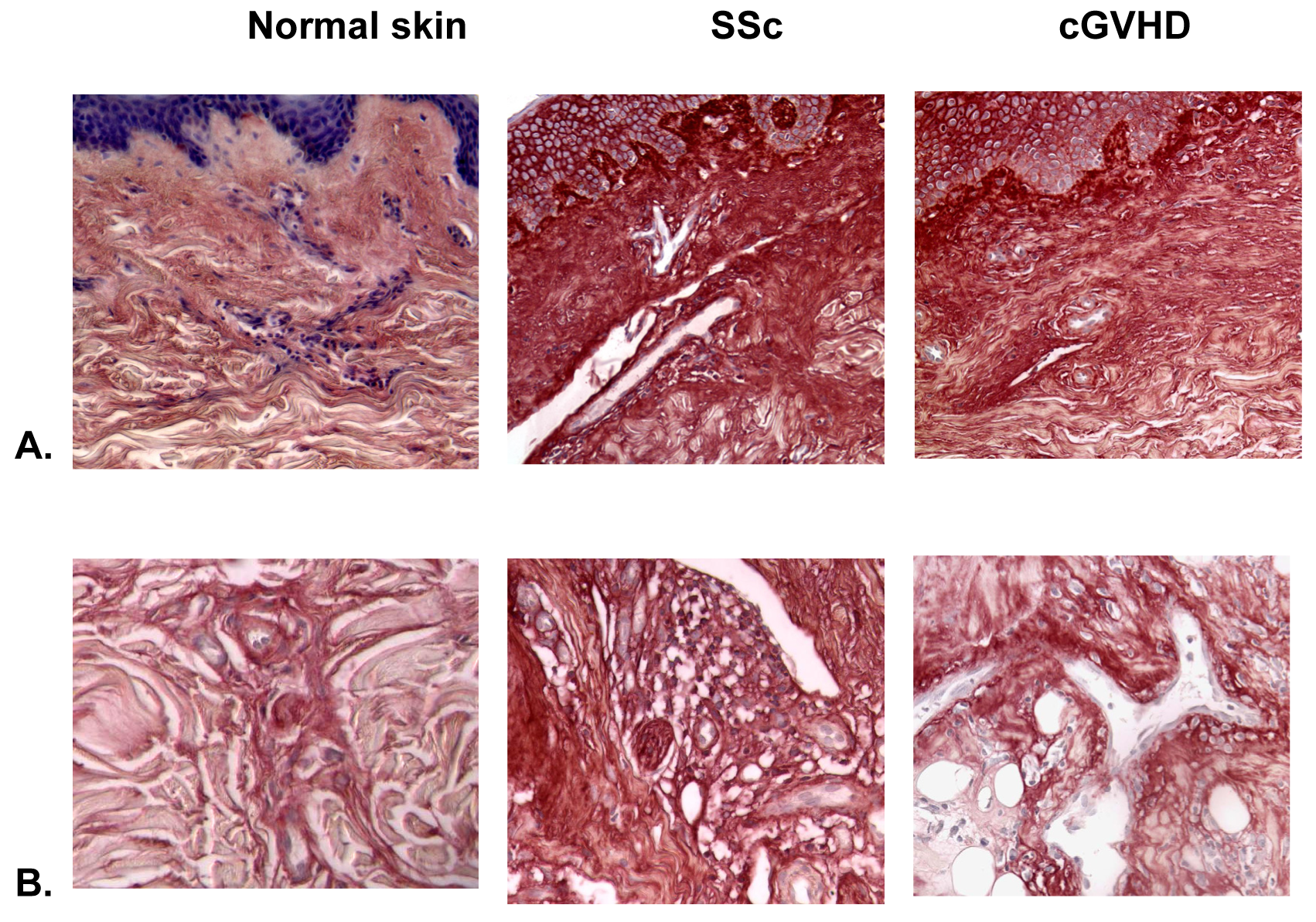

Supplement: Figure S1 — Histochemistry of scleroderma dyregulation of matix molecules A. Increased hyaluronan is present in Ssc and GVHD in the A dermal matrix and in B. areas of microvascular proliferation/cellularity. (1.72 MB TIF) [file pone.0006203.s001.tif]

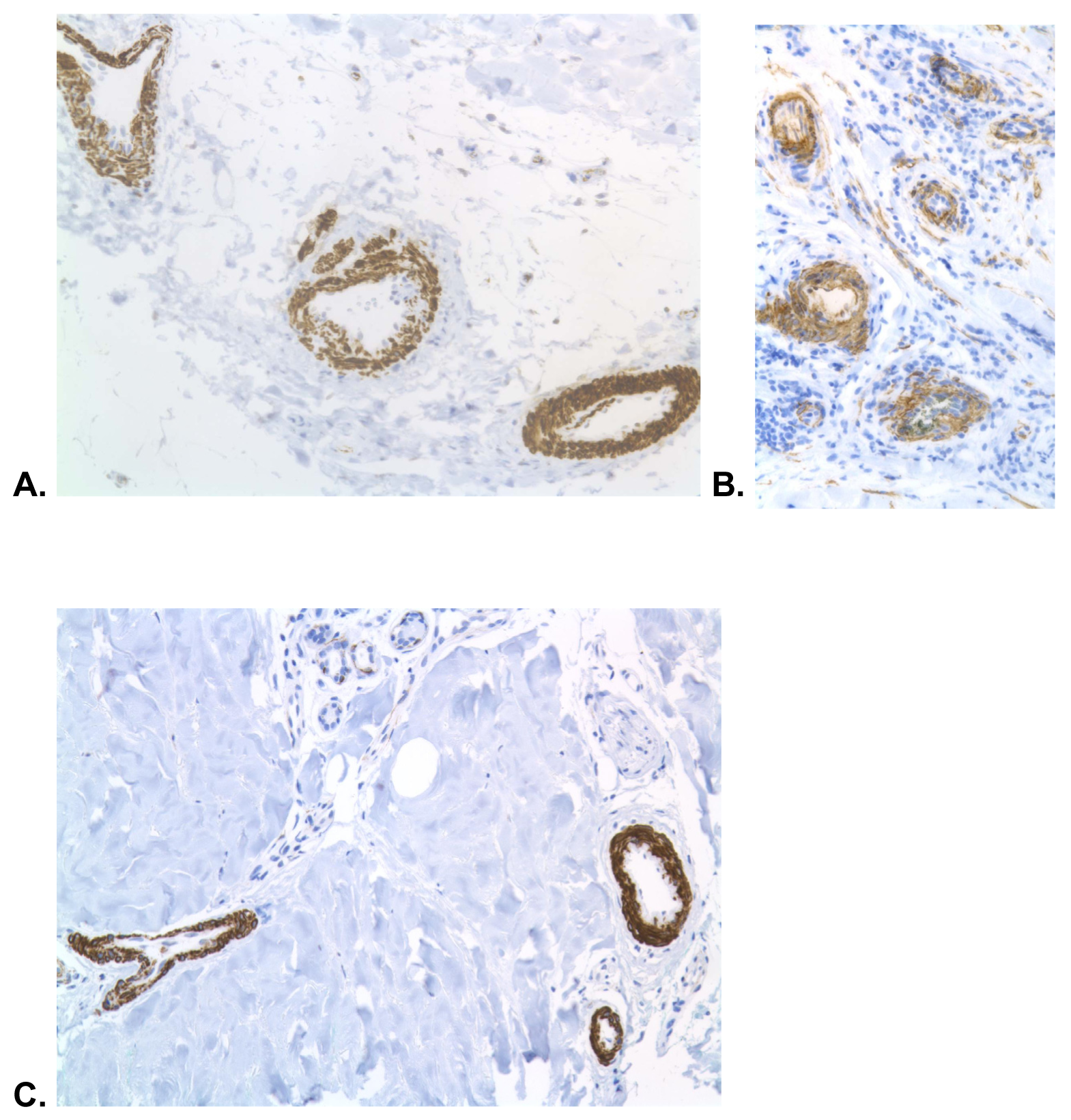

Supplement: Figure S2 — Intimal hyperplasia or smooth muscle hyperplasia in SSc and c-GVHD A. lichenoid c-GVHD SMMHC antibody depicts multiple layers of smooth muscle in skin vessels. No such vessels were found in any of the many full thickness biopsies of normal controls skin. B. Sclerotic c-GVHD stained with SMA, shows both larger and smaller vessels with multiple layers of smooth muscle cells, with scattered myofibroblasts and increased cellularity highlighting the inflammatory nature of c-GVHD C. SSC stained with an antibody to SMMHC shows thickened vessel walls around swollen endothelial layer (1.80 MB TIF) [file pone.0006203.s002.tif]

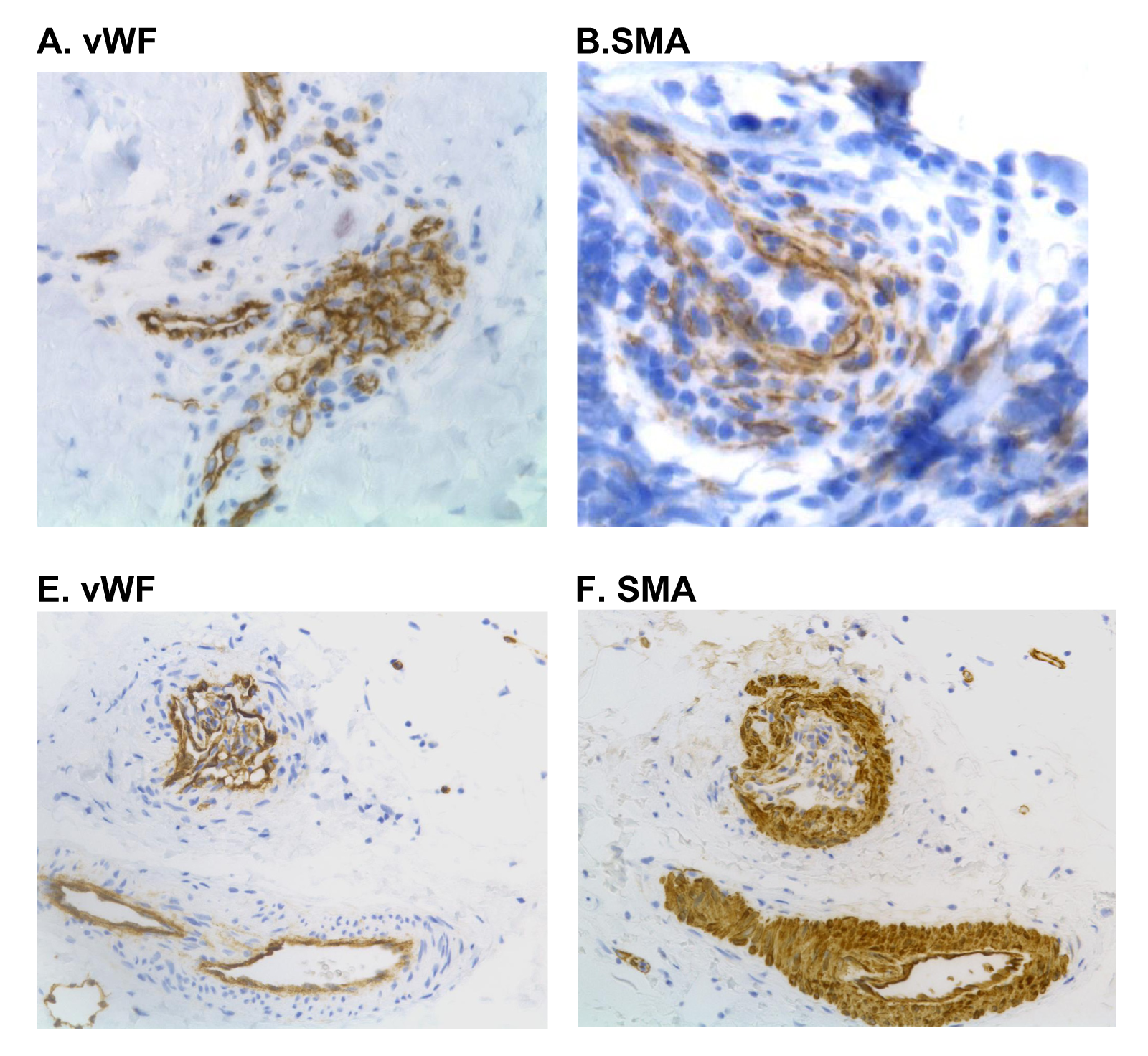

Supplement: Figure S3 — Endothelial markers in clumps of microvascular proliferative formations in GVHD Sclerotic cGVHD with areas of microvascular proliferation as defined by A. vWF B. SMA similar formations were seen in lichenoid cGVHD as defined by E. vWF and F. SMA similar appearing structures in SSc did not have endothelial markers present in the cells G. VE cadherin is shown with H. CD31, positive cells are sparse in these areas, although multiple lumens are present (2.56 MB TIF) [file pone.0006203.s003.tif]
