# Supplementary material for: Cutaneous Chronic Graft-Versus-Host Disease Does Not Have the Abnormal Endothelial Phenotype or Vascular Rarefaction Characteristic of Systemic Sclerosis
Source: PLoS One. 2009 Jul 9;4(7):e6203. doi: 10.1371/journal.pone.0006203 (PMC2705674; doi:10.1371/journal.pone.0006203)
Supplement: Table S1 — (0.07 MB DOC) [file pone.0006203.s004.doc]

| Supplemental Table 1 Normal controls | | | | | |
| --- | --- | --- | --- | --- | --- |
| Patient# | Age | Sex | Disease  Type | Biopsy  Location | Disease  Duration |
| 1 | un | un | N/A | scalp | N/A |
| 2 | un | un | N/A | un | N/A |
| 3 | un | un | N/A | scalp | N/A |
| 4 | 45 | F | N/A | forearm | N/A |
| 5 | un | un | N/A | un | N/A |
| 6 | un | un | N/A | Scalp | N/A |
| 7 | un | un | N/A | un | N/A |
| 8 | un | un | N/A | un | N/A |
| 9 | un | un | N/A | scalp | N/A |
| 10 | un | un | N/A | un | N/A |
| 11 | un | un | N/A | un | N/A |
| 12 | un | un | N/A | breast | N/A |
| 13 | un | un | N/A | un | N/A |
| 14 | un | un | N/A | abdomen | N/A |
| 15 | un | un | N/A | breast | N/A |
| 16 | un | un | N/A | un | N/A |
| 17 | un | un | N/A | un | N/A |
| 18 | un | un | N/A | Forearm | N/A |
| 19 | un | un | N/A | Forearm | N/A |
| 20 | un | un | N/A | Forearm | N/A |
| 21 | un | un | N/A | Forearm | N/A |
| 22 | un | un | N/A | Forearm | N/A |
| 23 | un | un | N/A | Forearm | N/A |
| 24 | un | un | N/A | Forearm | N/A |
| 25 | un | un | N/A | Forearm | N/A |
| 26 | un | un | N/A | un | N/A |
| 27 | un | un | N/A | un | N/A |
| 28 | un | un | N/A | un | N/A |
| 29 | un | un | N/A | un | N/A |
| 30 | un | un | N/A | un | N/A |
| 31 | un | un | N/A | thigh | N/A |
| Abbreviations Un=unavailable at this time N/A=nor applicable | | | | | |
