# Supplementary material for: Cutaneous Chronic Graft-Versus-Host Disease Does Not Have the Abnormal Endothelial Phenotype or Vascular Rarefaction Characteristic of Systemic Sclerosis
Source: PLoS One. 2009 Jul 9;4(7):e6203. doi: 10.1371/journal.pone.0006203 (PMC2705674; doi:10.1371/journal.pone.0006203)
Supplement: Table S2 — (0.06 MB DOC) [file pone.0006203.s005.doc]

| Supplemental Table 2 Scleroderma patients | | | | | |
| --- | --- | --- | --- | --- | --- |
| Patient# | Age | Sex | Disease  Type | Biopsy  Location | Disease  Duration |
| 1a  1b  1c | 47  48  51 | F | Diffuse | Forearm  “  “ | 1 year  2 years  5 years |
| 2a  2b  2c  2d | 44  46  “  48 | F | Diffuse | Forearm  “  Back  Forearm | 1 year  3 years  “  5 years |
| 3 | 47 | M | Diffuse | Forearm | 1 year |
| 4a  4b | 44  “ | F | Diffuse | Forearm  Upper Arm | 1 year  1 year |
| 5 | un | M | Diffuse | un | un |
| 6 | un | un | Diffuse | Forearm | un |
| 7 | un | un | Diffuse | Forearm | un |
| 8 | un | un | Diffuse | Forearm | un |
| 9 | un | un | Diffuse | Forearm | un |
| 10 | un | un | Diffuse | Forearm | un |
| 11 | un | un | Diffuse | Forearm | un |
| 12 | un | un | Diffuse | Forearm | un |
| 13 | un | un | Diffuse | Forearm | un |
| 14 | un | un | Diffuse | Forearm | un |
| 15 | un | un | Diffuse | Forearm | un |
| 16 | un | un | Diffuse | Forearm | un |
| 17 | un | un | Diffuse | Forearm | un |
| 18 | un | un | Diffuse | Forearm | un |
| 19 | un | un | Diffuse | Forearm | un |
| 20 | un | un | Diffuse | Forearm | un |
| 21 | un | un | Diffuse | Forearm | un |
| 22 | un | un | Diffuse | Forearm | un |
| 23 | un | un | Diffuse | Upper Arm | un |
| Abbreviations un=unavailable at this time | | | | | |
