# Supplementary material for: Cutaneous Chronic Graft-Versus-Host Disease Does Not Have the Abnormal Endothelial Phenotype or Vascular Rarefaction Characteristic of Systemic Sclerosis
Source: PLoS One. 2009 Jul 9;4(7):e6203. doi: 10.1371/journal.pone.0006203 (PMC2705674; doi:10.1371/journal.pone.0006203)
Supplement: Table S3 — (0.04 MB DOC) [file pone.0006203.s006.doc]

| Supplemental Table 3 Antibodies used in this study | | |
| --- | --- | --- |
| **Antibody abbreviation** | Full name Function | **Manufacturer/ Clone/cat#**  **Pretreatment**  **Dilution**  **Secondary**  **Detection** |
| **CD31^a,b^** | PECAM1 implicated in several functions, including transendothelial migration of leukocytes, angiogenesis, and integrin activation. | Cell Marque/1A10 DAKO Target Retrival Solution 1:50 DAKO Envision Dual Link Polymer |
| **vWF^a,b^** | Von Willebrand Factor In endothelial cells that produce VWF, the factor is stored in so-called Weibel-Palade bodies. The factor released from these bodies binds particularly avidly to the extracellular matrix. Following secretagogue stimulation, Weibel-Palade bodies undergo exocytosis and release long VWF filaments, averaging 100 micrometers, that capture platelets along their length. Subsequent activation and aggregation of platelets cause the formation of a hemostatic plug | DAKO / A0082 DAKO Target Retrival Solution 1:4000 DAKO Envision Dual Link Polymer |
| **VEC^a^** | VE Cadherin vascular endothelial cadherin plays a key role in angiogenesis and in vascular permeability. Regulates cytoskeletal tension, internalization of the VEGF receptor. The regulation of its biological activity may be a central mechanism in normal or pathological angiogenesis. | Novocastra/BV6  Citrate/heat  1:100  Vector Biomouse Elite  DAB |
| **ASMA ^a,b^** | smooth muscle actin, an isoform typical of smooth muscle cells (SMC) and present in high amounts in vascular SMC and pericytes, localized in microfilament bundles, strengthening the assumption that it is the functional isoform in these cell types and supporting the assumption that pericytes exert contractile functions. | DAKO /1A4  Pronase or DAKO Target Retrival Solution  1:250  Mouse/ Envision  DAB or DAKO Envision Dual Link Polymer |
| SMMHC**^a^** | smooth muscle myosin heavy chain The smooth muscle myosin heavy chain (SM-MHC) gene encodes a major contractile protein whose expression exclusively marks the smooth muscle cell (SMC) lineage. | DAKO /M3558  Pronase  1:200  Mouse envision  DAB |
| Ha^c^ | Hyaluronan serves a variety of functions, including space filling, lubrication of joints, and provision of a matrix through which cells can migrate. | HABR  None  4ug/ml  ABC Elite  Nova Red |
| a=IHC performed at Phenopath Laboratories  b=IHC performed at SCCA lab  c=IHC performed at Wight Lab | | |
